# The Ex-utero intrapartum treatment procedure: a narrative review

**DOI:** 10.3389/fped.2025.1601963

**Published:** 2025-07-17

**Authors:** Michele Gaffuri, Genny Raffaeli, Elena Emilia Bullejos Garcia, Giuseppe Perugino, Ottavio Cassardo, Nicola Persico, Mariarosa Colnaghi, Felipe Garrido, Eduardo Villamor, Irene Cetin, Monica Fumagalli, Lorenzo Pignataro, Giacomo Cavallaro

**Affiliations:** ^1^ Department of Otolaryngology and Head and Neck Surgery, Fondazione IRCCS Ca’ Granda Ospedale Maggiore Policlinico, Milan, Italy; ^2^ Department of Clinical Sciences and Community Health, Dipartimento di Eccellenza 2023-2027, University of Milan, Milan, Italy; ^3^ Neonatal Intensive Care Unit, Fondazione IRCCS Ca’ Granda Ospedale Maggiore Policlinico, Milan, Italy; ^4^ Pediatric Anesthesia and Resuscitation Unit, Fondazione IRCCS Ca’ Granda Ospedale Maggiore Policlinico, Milan, Italy; ^5^ Department of Obstetrics and Gynecology, Fondazione IRCCS Ca’ Granda Ospedale Maggiore Policlinico, Milan, Italy; ^6^ Prenatal Diagnosis and Fetal Surgery Unit, Fondazione IRCCS Ca’ Granda Ospedale Maggiore Policlinico, Milan, Italy; ^7^ Neonatal Intensive Care Unit, Clínica Universidad de Navarra, Madrid, Spain; ^8^ Division of Neonatology, MosaKids Children’s Hospital, Maastricht University Medical Center (MUMC+), Research Institute for Oncology and Reproduction (GROW), Maastricht University, Maastricht, Netherlands

**Keywords:** Ex utero intrapartum treatment, EXIT, newborn, airway obstruction, tracheal occlusion, congenital neck masses, vascular abnormalities, lymphatic malformations

## Abstract

The “Ex Utero Intrapartum Treatment” (EXIT) procedure is a specialized surgical technique used during cesarean delivery to perform life-saving fetal interventions while maintaining placental circulation. By preserving feto-placental gas exchange, EXIT enables the treatment of severe conditions such as predictable severe breathing difficulties at birth. EXIT's origins date back to removing tracheal occlusion devices used for congenital diaphragmatic hernias. It has since expanded to treat conditions such as congenital high airway obstruction syndrome and airway compression by masses. Despite the risks of adverse maternal and fetal events, it shows high perinatal survival rates. The success of EXIT depends on an accurate prenatal diagnosis through fetal ultrasound and magnetic resonance imaging. Anesthetic management differs from standard cesarean sections, balancing the need for uterine relaxation and avoiding maternal-fetal risks. Inhaled anesthetics are preferred, although recent studies suggest the potential of neuraxial anesthesia combined with tocolytics. Although the EXIT procedure can be performed safely in specialized centers, it does carry risks for both the mother and the fetus. Neonatal mortality and complications vary depending on indications and postnatal management. Research and clinical practice must advance to improve safety and efficacy.

## Key points

• Ex utero intrapartum treatment (EXIT) is a specialized surgical procedure performed during a cesarean section that facilitates fetal interventions. At the same time, the fetus remains connected to the placenta, allowing for gas exchange through the fetoplacental circulation.
• Maintaining fetoplacental circulation enables effective management of specific medical situations in which the fetus may experience significant respiratory difficulties at birth.
• Indications for EXIT include conditions such as high airway obstruction, compressive cervical or thoracic masses requiring removal, and other scenarios where postnatal respiratory compromise is anticipated, especially when intubation is difficult or normal cardiorespiratory function is altered.
• The success of the EXIT procedure relies on the collaboration of a highly specialized interdisciplinary team, which should include obstetricians, pediatric surgeons, anesthetists, neonatologists, otolaryngologists, specialized nurses, and, when necessary, cardiac surgeons and perfusionists for extracorporeal membrane oxygenation (ECMO).

## 1 Introduction

**“**Ex utero intrapartum treatment” (EXIT) is a highly specialized surgical approach used in obstetrics and neonatology (1). This procedure, performed during cesarean delivery, enables interventions on the fetus while still connected to the placenta, utilizing feto-placental circulation for gas exchange (1). This approach is valuable in cases where the fetus experiences significant respiratory distress at birth (2–4).

Norris et al. first described the operation on placental support (OOPS) in 1989, while the term “EXIT procedure” was later coined by Mychaliska et al. in 1997, outlining a systematic approach for managing complex fetal airways (5, 6). Since then, EXIT has become a revolutionary method adopted worldwide for managing congenital airway anomalies that would otherwise be incompatible with life (7, 8). This procedure was initially employed to remove tracheal occlusion devices (clips, plugs, or balloons) to decrease pulmonary hypoplasia in congenital diaphragmatic hernias. Since then, its application has expanded to include other conditions, such as congenital high airway obstruction syndrome (CHAOS) or airway compression caused by intra- or extra-thoracic masses (9–11).

These obstructions can be critically life-threatening or result in prolonged hypoxia with long-term neurodevelopmental consequences (9, 11–14).

In the contemporary clinical practice, the EXIT procedure is indicated in three main scenarios:

1. Upper Airway Obstruction: When an obstruction prevents the newborn from establishing a clear airway.
2. Cervical or Thoracic Masses: To remove masses that compress vital neck or chest structures.
3. Postnatal Respiratory Distress: Cases where immediate respiratory support is needed.

These are further reclassified, and in this context, we will explore specific variations of the EXIT procedure (Table 1) (12, 15):

1. “EXIT to Airways”: Securing the airway in obstruction cases.
2. “EXIT to Resection”: Removing compressive masses.
3. “EXIT to ECMO”: Providing ECMO support for severe respiratory or cardiac failure.
4. “EXIT to Separation”: Facilitating surgical separation of conjoined twins or complex anomalies.

**Table 1:** Indication for Ex utero intrapartum treatment (EXIT).

EXIT procedure	Site of obstruction	Pathologies	Grade of indication
EXIT to Airways	Extrinsic	Teratoma (cervical, pharyngeal, epignathus) Lymphatic/vascular malformation Congenital giant ranula Severe micrognathia	Mandatory
			Mandatory
			Mandatory
			Elective or adjunctive
	Intrinsic	Congenital intrinsic high airway obstruction syndrome (CHAOS)	Mandatory
	Iatrogenic	Iatrogenic tracheal clamping or balloon tracheal occlusion in patients with congenital diaphragmatic hernia	Mandatory
EXIT to Resection		Thoracic/mediastinal masses Cystic adenomatoid lung malformations (CPAM) Bronchopulmonary sequestration Fetal lobar interstitial tumor Mediastinal teratoma	Elective or adjunctive
			Elective or adjunctive
			Elective or adjunctive
			Elective or adjunctive
			Elective or adjunctive
EXIT to ECMO		Severe congenital heart disease Severe congenital diaphragmatic hernia	Elective or adjunctive
			Elective or adjunctive
EXIT to separation		Siamese twins	Elective or adjunctive

This narrative review aims to provide an overview of the indications, techniques, potential complications, and outcomes associated with the EXIT airway management procedure, focusing on treating airway obstruction and compressive masses.

### 1.1 Upper airway obstructions – “EXIT to airways” and “EXIT to resection”

Obstructive lesions can be extrinsic, such as compressions by a cervical, pharyngeal, or thoracic mass; intrinsic, such as CHAOS; or iatrogenic, such as occurs with tracheal obstruction through the insertion of a tracheal plug/balloon during fetal life to treat severe congenital diaphragmatic hernia (8, 9, 15–18).

#### 1.1.1 Lymphatic malformations

Lymphatic malformations are the second most common cause of neonatal soft tissue tumors, following hemangiomas, and are often linked to the EXIT procedure (19, 20). These malformations develop during the second and third trimesters of pregnancy due to abnormal embryological processes affecting the lymphatic system, such as isolation of the original lymph sac, disturbances in lymphatic and venous flow, or abnormal hyperplasia of lymphatic tissue (20, 21).

Lymphatic malformations are classified into macrocystic (>2 cm), microcystic (<2 cm), or mixed types. Macrocystic lesions tend to compress surrounding tissues, while microcystic lesions infiltrate them. Postnatally, macrocystic lymphatic malformations appear as soft masses, whereas microcystic ones are more complex and infiltrative (22).

The severity and prognosis of lymphatic malformations in the head and neck depend on their position relative to the hyoid bone (supra- or sub-hyoid) and laterality (unilateral or bilateral). Bilateral forms in both positions are linked to a higher risk of complications (23).

In addition, growth in the oral or pharyngolaryngeal cavities can lead to issues with swallowing, speech, and temporomandibular joint disorders (22).

#### 1.1.2 Teratomas

Teratomas are germ cell tumors that usually develop during the fourth or fifth week of gestation and arise from the three embryonic layers: ectoderm, mesoderm, and endoderm (24). These ectopic germ cells proliferate and differentiate into either mature tissue (mature teratoma) or fetal tissue (immature teratoma). The incidence of these congenital tumors is approximately 1 in 20,000 to 40,000 live births. Although they can originate in various anatomical regions, such as the sacrococcygeal area, reproductive organs, anterior mediastinum, and retroperitoneum, 6% of these tumors are in the head and neck region, often developing from the thyrocervical area of the palate or nasopharynx (25). Although primarily solid, they typically contain cysts and calcifications (26–28).

Epignathus, a rare oropharyngeal teratoma from Rathke's pouch, occurs in about 1 in 35,000 to 200,000 live births. While usually benign, its size and location can cause airway obstruction and difficulty swallowing (29).

#### 1.1.3 Congenital giant ranula

Congenital giant ranula is a rare condition in neonates, presenting as a large cyst in the floor of the mouth due to atresia or failure to channel the salivary ducts of the sublingual or submandibular gland. It affects about 0.7% of infants (30, 31). The condition arises from the rupture of the salivary gland's excretory duct, leading to an accumulation of mucinous secretion and local inflammation, resulting in pseudocyst formation. Treatment options include observation, aspiration, marsupialization, and surgical excision, with the latter being the most effective for preventing recurrence (32). In rare cases, the cyst can obstruct the airway, necessitating the EXIT procedure, which involves decompressing the ranula for safe intubation (4, 12, 15). Literature on this condition and the EXIT procedure is limited (30, 32).

#### 1.1.4 Severe micrognathia

Severe micrognathia, caused by mandibular hypoplasia, can cause glossoptosis that obstructs the upper aero-digestive tract, complicating endotracheal intubation (33). Although it could be isolated, it often accompanies conditions like otocephaly, dysgnathia, Pierre Robin sequence, and Treacher-Collins, Nager, or velocardiofacial syndromes (34–36). Although many infants with micrognathia do not experience airway obstruction, severe cases have a high risk of respiratory failure, with a survival rate of 10%–20% (37). Less severe micrognathia presents with obstruction in 54%–88% of patients, with 42%–57% requiring intubation or tracheostomy (38, 39). Recent findings suggest intubation is only necessary in 25% of patients, with a mortality rate of 0%–6%. Consequently, the EXIT procedure is reserved for severe cases of mandibular hypoplasia, where difficult intubation or emergency tracheostomy is anticipated (39–41).

Diagnosing the degree of airway obstruction in severe micrognathia is challenging. A 2021 study by Tay et al. demonstrated that a mandibular index below the 5th percentile or abnormal amniotic fluid index accurately predicts the severity of airway obstruction (40). Therefore, if the mandibular index, which is calculated by dividing the anteroposterior diameter of the mandible by the biparietal diameter and then multiplying by 100, falls below the 5th percentile and there are signs of obstruction, such as an absent ultrasound-visible stomach, polyhydramnios, or glossoptosis, an EXIT procedure should be considered (39, 42).

#### 1.1.5 Congenital high airway obstruction syndrome (CHAOS)

Congenital high airway obstruction syndrome is a rare condition resulting from the failed canalization of the upper airways during fetal development, typically around the 10th week of gestation. This condition includes anomalies such as laryngeal atresia, laryngeal cysts, tracheal agenesis, and laryngeal webs, with laryngeal atresia being the most common cause.

In 1994, Hedrick et al. introduced the term “CHAOS” to describe ultrasound findings in four fetuses with upper airway obstruction considered incompatible with survival (43). The exact incidence of CHAOS remains unknown due to its rarity (44). In some cases, spontaneous reductions of airway obstruction can occur in the third trimester of pregnancy. Complete obstruction is often associated with tracheoesophageal fistula (37, 45). Without intervention, CHAOS usually leads to intrauterine or neonatal death (43, 46).

During fetal life, this condition prevents normal lung fluid outflow, leading to increased intrathoracic pressure, pulmonary hyperplasia, heart failure, and fetal hydrops (45, 47, 48). Hyperechogenic lungs, flattening or inversion of the diaphragmatic domes, and dilation of the distal airway represent the triad of radiological diagnostic signs of the syndrome (49). CHAOS may be linked to various congenital anomalies, such as esophageal atresia, imperforate anus, renal agenesis, ambiguous genitalia, hydrocephalus, anophthalmia, spinal abnormalities, and syndactyly. Additionally, it can be associated with genetic syndromes like Fraser syndrome and Fragile X syndrome, which may increase the risk of fetal death (4, 50). The EXIT procedure has significantly reduced morbidity and mortality by enabling the safe placement of a tracheostomy while maintaining fetoplacental circulation. This approach has transformed the management of CHAOS, offering a lifeline for affected fetuses (51–54).

#### 1.1.6 Iatrogenic tracheal stenosis

Historically, the EXIT procedure was used to remove tracheal clips placed in fetuses with congenital diaphragmatic hernia (CDH). Today, clips have been replaced by the fetoscopic insertion of a tracheal balloon between the 27th and 29th weeks of gestation, with fetoscopic removal occurring around 34 weeks (55). However, the EXIT procedure can still be utilized in complex cases, such as when spontaneous labor occurs before the second fetoscopy or when the fetal position makes the fetoscopic approach impractical. Recently, researchers developed a magnetic-controlled tracheal occlusion balloon that can be deflated using a magnetic field. This eliminates the need for a second fetoscopy or an emergency EXIT procedure (56–58)*.*

### 1.2 Cervical or thoracic compressive mass - “EXIT to resection”

Cervical and thoracic compressive masses, such as teratomas, lymphatic malformations, congenital pulmonary airway malformation, bronchogenic cysts, and bronchopulmonary sequestration, can profoundly influence fetal development. These conditions may result in serious complications, including tracheal obstruction, mediastinal shift, pulmonary hypoplasia, hydrops, and hypoxia at birth (59). While there is potential for many thoracic masses to regress spontaneously during the third trimester, some may continue to expand, necessitating medical intervention to avert perinatal mortality (60, 61).

The EXIT procedure represents a crucial intervention for neonates with large thoracic masses who are at high risk of airway obstruction and hypoxia at birth. In this particular scenario, the primary goal of the EXIT procedure is to remove the mass, taking advantage of placental oxygenation surgically (11, 61). Therefore, EXIT reduces the need for emergency postnatal surgery and enhances long-term pulmonary function and survival, even though definitive postnatal resection of thoracic masses may still be necessary later (59, 60, 62).

### 1.3 Other indications for EXIT - “EXIT to ECMO” and “EXIT to separation”

In cases of severe cardiothoracic malformations, securing the airway through an EXIT procedure may not always be feasible. In such scenarios, implementing ECMO effectively during the procedure can facilitate a more adaptable and gradual postnatal treatment plan (63). Although this approach has shown success, cases involving significant cervical masses remain rare and are associated with high mortality due to the need for anticoagulation and central cannulation via sternotomy (59–67, 61). Therefore, combining EXIT with ECMO should only be considered a last resort after evaluating all other possible measures.

The use of EXIT and ECMO for severe CDH has been described, but the literature remains controversial (65). Studies indicate that survival rates, as well as pulmonary, cardiac, and psychomotor development, in neonates with severe CDH who received ECMO via EXIT were comparable to those who received ECMO postnatally (65).

The EXIT procedure has also been described for the separation of conjoined twins, particularly thoracopagus twins with congenital heart defects (68, 69). However, the literature is divided on this approach, as many authors suggest that postnatal delivery, imaging studies, and controlled tissue expansion yield better outcomes than separation during EXIT (4).

### 1.4 Prenatal diagnosis and EXIT timing

The success of the EXIT procedure is closely tied to accurate and timely prenatal diagnosis. As such, prenatal evaluation plays a critical role in this process. When a malformation involving the fetal high airway is detected or suspected, the patient must be promptly referred to a specialized center with expertise in fetal ultrasound and MRI (70–79). Additionally, collaboration with a medical genetics center experienced in conducting specific genetic investigations is essential to ensure a comprehensive diagnosis (76).

The optimal timing for conducting an EXIT procedure is generally established between 35 and 37 weeks of gestation. This timeframe permits adequate fetal lung maturation while minimizing potential risks for both the mother and the infant (12, 62). A systematic review of EXIT procedures demonstrated an average gestational age of 35.1 weeks, underscoring the necessity of balancing neonatal survival and maternal safety (12).

In severe airway obstruction or polyhydramnios, it may be necessary to consider an earlier intervention, potentially as early as 30–34 weeks. However, such early procedures are associated with an increased risk of neonatal morbidity (80). Consequently, performing EXIT procedures at gestational ages greater than 35 weeks is advisable, as this approach mitigates the risk of preterm complications while facilitating safe airway management (62).

### 1.5 Anesthesiologic considerations

Anesthesia management during the EXIT procedure presents unique challenges compared to a conventional cesarean section (81). A primary objective during EXIT is to achieve optimal uterine relaxation, intending to reduce the risk of placental abruption while ensuring the maintenance of placental circulation (1, 82).

Goals for the EXIT procedure include providing adequate general anesthesia to the mother, maximizing uterine relaxation for fetal head expulsion, ensuring uterine blood flow for fetal oxygenation, and minimizing fetal movement during surgery in EXIT to resection (82).

General anesthesia is typically preferred as it allows for better control of inhalation agents, although maternal factors can influence anesthesia choice (81–92).

Achieving uterine relaxation involves not just tocolytics, such as beta-mimetic drugs, oxytocin receptor antagonists, and cyclooxygenase inhibitors. It may also include high concentrations of inhalational anesthetics, which can cause maternal hypotension and uteroplacental hypoperfusion. Alterations in maternal cardiorespiratory function may further complicate the situation, potentially leading to hypoxia (82, 93–98).

In addition, inhalational anesthetics can negatively impact fetal cardiovascular homeostasis (94, 99–101). Recent studies suggest combining neuraxial anesthesia with tocolytics could improve conditions during EXIT, but this approach requires careful patient selection since the mother remains alert (81, 88, 93). Neuraxial anesthesia can reduce bleeding and transfusion needs, but it is not suitable for long-term placental bypass cases (81, 88).

Inhalational agents like desflurane, sevoflurane, and isoflurane are commonly chosen to promote uterine relaxation (91, 102–105). Intravenous agents such as propofol and remifentanil have also shown potential as alternatives (91). Fetal anesthesia should be considered when a mother undergoes neuraxial anesthesia. This entails the intramuscular administration of a combination of medications, specifically fentanyl, atropine, and a drug for neuromuscular blockage (106).

Monitoring is crucial during the procedure. Maternal blood pressure, oxygen saturation, end-expiratory carbon dioxide levels, and fetal preductal oxygen saturation help detect potential complications (1, 4).

### 1.6 Obstetrical considerations

Both maternal health and uteroplacental stability must be systematically evaluated and prioritized throughout the EXIT procedure (107, 108). Effective management of polyhydramnios is critical during the planning phase of the EXIT procedure to reduce maternal-fetal complications. In cases of severe polyhydramnios, preoperative amnioreduction is recommended to alleviate uterine overdistension, which is a recognized risk factor for placental abruption and intraoperative uterine rupture (6, 8, 109–111). Furthermore, it is essential to assess both placental location and fetal head position, as these factors are crucial for guiding the uterine incision and minimizing the risk of hemorrhagic complications (112–114). Patients are typically positioned with a left lateral tilt to prevent inferior vena cava compression (115). The uterine incision is generally executed using a Pfannenstiel or low midline approach. Accurate and precise execution of the incision is paramount in order to reduce myometrial bleeding and minimize associated risks (83). In urgent situations where amnioreduction is either contraindicated or not feasible, the risk of placental abruption can be effectively reduced through controlled amniotic drainage (116, 117). Following the myometrial incision, the procedure necessitates meticulous preparation and exposure of an intact amniotic sac. Subsequently, multiple small punctures are performed in the sac, facilitating gradual uterine decompression before the delivery of the fetal head (118). The hysterotomy is strategically positioned slightly above the lower uterine segment to optimize space for fetal head extraction while avoiding highly vascularized areas (107). To prevent uterine wall bleeding, the free uterine margin can be manually sutured or stapled before the incision of the amniotic sac (4, 87, 96, 119). Incisions may be extended to ensure safe fetal exposure when addressing large neck masses (85, 87, 96). The umbilical cord may be clamped upon establishing the neonatal airway and initiating ventilation. The moment of birth for the newborn is specifically defined as the time of umbilical cord clamping, rather than at the point of head emergence (120). Fetal malpresentation poses a significant challenge during an EXIT procedure. In cases of a breech or transverse lie, the uterine incision must be tailored to ensure optimal exposure of the presenting part while safeguarding placental integrity. A high transverse or classical vertical incision may be appropriate, provided the placenta is not previa and its location is favorable (10). In cases of breech presentation, an alternative approach is the internal cephalic version, which aims to facilitate the delivery of the fetal head. While the execution of this maneuver can be complex, coexisting polyhydramnios may provide adequate space, thus simplifying the process. Conversely, significant challenges arise when the EXIT procedure is further complicated by placenta previa, necessitating careful management to ensure that the uterine incision does not transect the placenta, as this could result in catastrophic hemorrhage. One strategy that has been proposed for addressing cases of placenta accreta spectrum involves the exteriorization of the gravid uterus, followed by a fundal hysterotomy. Each step of this intricate procedure must be verified through real-time intraoperative ultrasound to accurately assess the placenta's position (121). Attention must be paid to the potential resultant iatrogenic angulation of the uterine arteries, as this condition can compromise placental perfusion, thereby necessitating more prompt management of the fetal airway. It is crucial to anticipate intraoperative complications, such as uterine hemorrhage, and it is recommended to ensure the availability of blood transfusion capabilities throughout the procedure (122). To mitigate the risk of postpartum hemorrhage, especially following prolonged uterine manipulation with sustained placental perfusion, the administration of prophylactic uterotonics, along with readiness for advanced interventions, is a critical component of care (120). Continuous instillation of lactated Ringer's solution aids in managing fluid loss throughout the procedure (123). Among secondary therapeutic strategies, the Bakri intrauterine balloon tamponade has demonstrated significant efficacy in controlling severe postpartum hemorrhage, achieving success rates of up to 84.5%, even in placenta accreta cases (124). Moreover, uterine devascularization techniques, such as bilateral uterine artery ligation, continue to represent a cost-effective and fertility-preserving option when bleeding persists or when balloon tamponade proves unsuccessful (125). Post-EXIT care repairs the uterus and ensures hemostasis, thereby minimizing the risk of future uterine rupture (87).

### 1.7 Neonatal and ENT considerations

**Figure 1:**
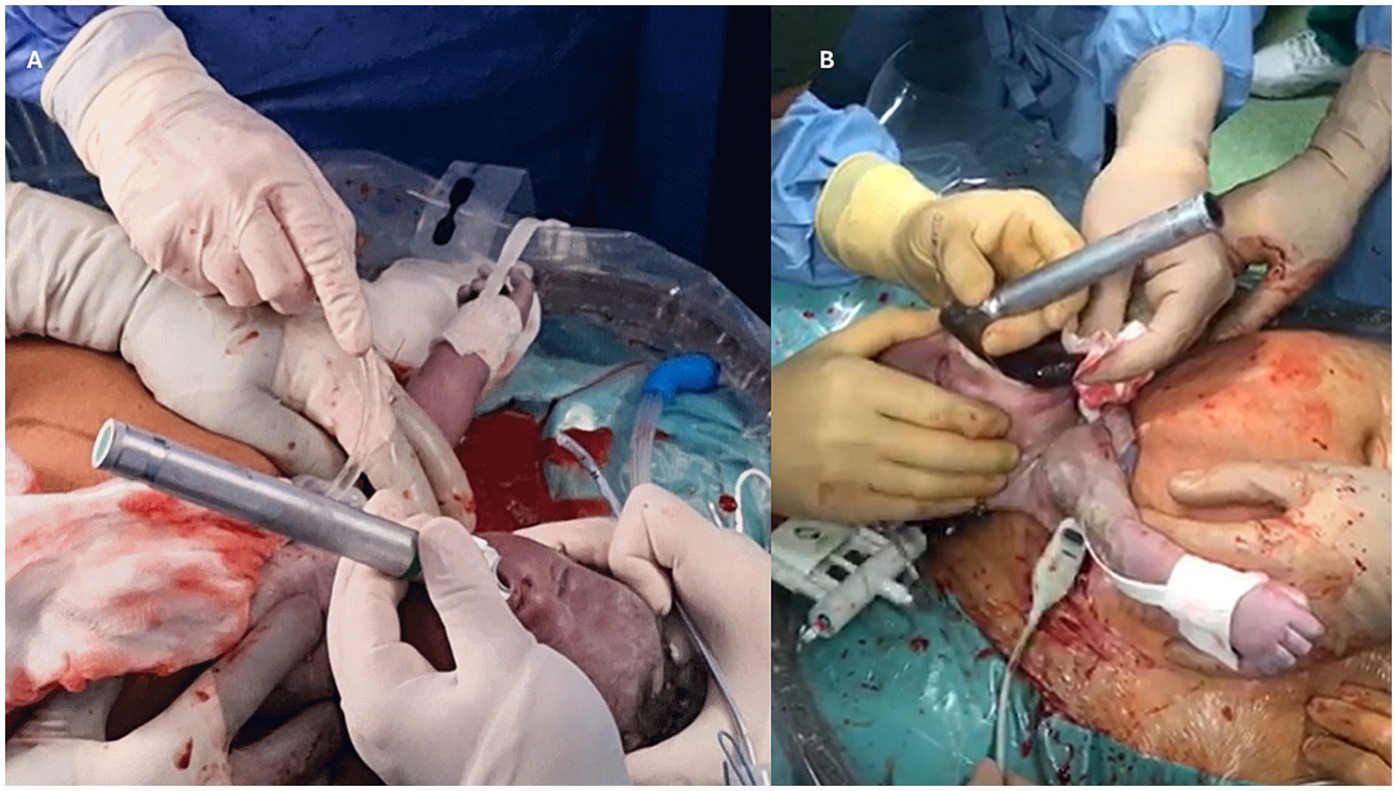
**(A,B)** direct laryngoscopy intubation of a fetus with a venous malformation of the tongue.

**Figure 2:**
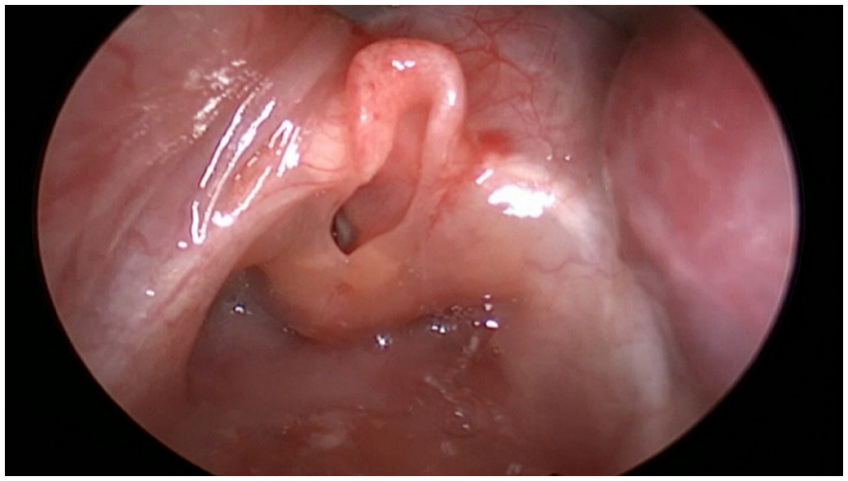
High-definition video laryngoscopy visualizing the fetal larynx during an EXIT delivery.

**Figure 3:**
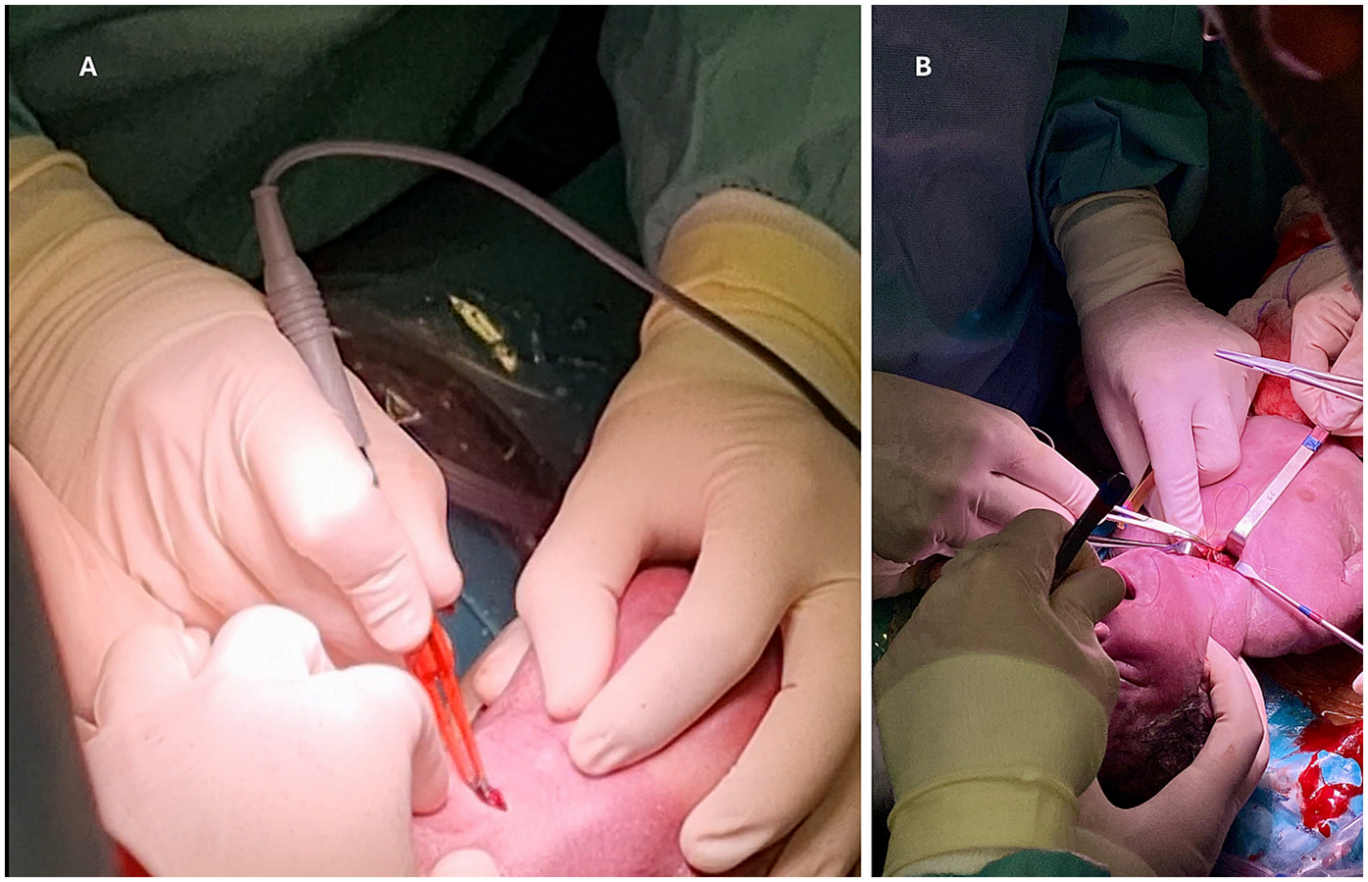
**(A,B)** surgical tracheotomy in EXIT of a fetus with laryngeal atresia.

During the EXIT procedure, neonatologists and/or ear, nose, and throat (ENT) surgeons focus on securing and maintaining a clear fetal airway while utilizing uteroplacental circulation to ensure optimal oxygenation (90). These teams must address possible airway obstructions that may arise from masses, congenital anomalies, or structural abnormalities. To manage these obstructions, a range of strategies may be employed, including direct laryngoscopy (Figure 1), rigid or flexible video laryngeal tracheoscopy (Figure 2), surgical tracheostomy (Figure 3), resection of cervical-facial or chest masses, and cannulation for ECMO. The approach will depend on the severity of the obstruction, starting with intubation attempts and escalating to more invasive measures, such as establishing a surgical airway as needed (126). Careful management of the fetal head and neck positions is crucial to optimize airway visualization and facilitate intervention. In cases where large cervical or thoracic masses are present, the ENT team plays a key role in planning for resection. The uterine incision may be extended to allow for adequate mass exposure while ensuring that placental gas exchange remains uninterrupted throughout the procedure. Special attention is given to avoiding pressure on critical structures, including the trachea, esophagus, and major blood vessels (119).

The degree of fetal exposure during the EXIT procedure varies based on intervention needs. Minimizing exposure is crucial to reduce risks like heat loss and umbilical cord issues (90). Continuous fetal monitoring is essential, including pulse oximetry and ultrasounds for blood flow and heart rate (10, 90, 120).

Fetal arterial saturation is monitored with a pulse oximeter on the right hand, typically within a normal range of 60%–70%, where values above 40% indicate adequate oxygenation. Intraoperative fetal echocardiography helps assess cardiovascular function and identify distress signs, necessitating timely interventions. In cases of fetal distress, blood gas samples can be drawn from umbilical vessels to guide treatment. Establishing intravenous access is also essential for fluids and medications (10, 90, 120). Additionally, capnography is used to rapidly confirm neonatal intubation by detecting exhaled carbon dioxide, proving faster and more reliable than colorimetric methods (127, 128). After delivery, the neonatology team transfers the infant to a nearby stabilization unit, prioritizing respiratory support and hemodynamic monitoring. Moreover, additional neonatal surgical interventions are evaluated at this time, and an operating block is prepared adjacent to the relevant team. Postnatal evaluation thoroughly assesses residual airway abnormalities, potential feeding difficulties, and long-term respiratory function (129).

### 1.8 Length of EXIT

The first documented EXIT procedure lasted 5–20 min and had high fetal morbidity (5). Recent advancements have increased uterine relaxation and prolonged uterine-placental circulation times, typically between 45 and 60 min, although the literature has reported uterine-placental circulation times as long as 150 min (6, 82, 86, 130, 131). A study by Bouchard et al. found an average of approximately 30.3 min for uteroplacental circulation in 31 EXIT cases (120).

The EXIT procedure is complex and requires careful consideration of maternal and fetal factors, appropriate anesthetic techniques, and monitoring for successful outcomes.

### 1.9 Maternal and fetal complications

The EXIT procedure carries more significant risks than conventional cesarean deliveries, particularly concerning bleeding, procedure duration, and scar-related complications that increase the risk of uterine rupture in future pregnancies. Approximately 6% of mothers may need a blood transfusion during the procedure. There is an estimated 11% risk of uterine rupture in subsequent pregnancies, which is similar to the risk associated with prenatal spina bifida surgery (132–134).

Fetal and neonatal mortality rates for the EXIT procedure range from 5% to 25%, while fetal complications occur in about 13% of cases (12) (135). Common causes of neonatal death include cardiopulmonary arrest, pulmonary hypoplasia, and hypoxia due to failed intubation or tracheostomy (80, 136, 137). Complications arising from the procedure are frequently associated with cardiovascular problems resulting from compression of the chest, neck, or umbilical cord, as well as the effects of anesthesia. Additionally, there have been documented cases of umbilical cord spasms in conjunction with temperature fluctuations (12, 109, 138).

Neurodevelopmental outcomes following EXIT procedures were generally positive, with most children demonstrating age-appropriate cognitive, language, and motor development. However, mild deficits were observed in 31% of language-related cases and 23% of motor skills cases, with no severe impairments reported (139). Improving resource allocation and implementing evidence-based protocols to enhance neurodevelopmental outcomes and reduce associated risks is crucial (129).

### 1.10 Multidisciplinary team and simulation

**Figure 4:**
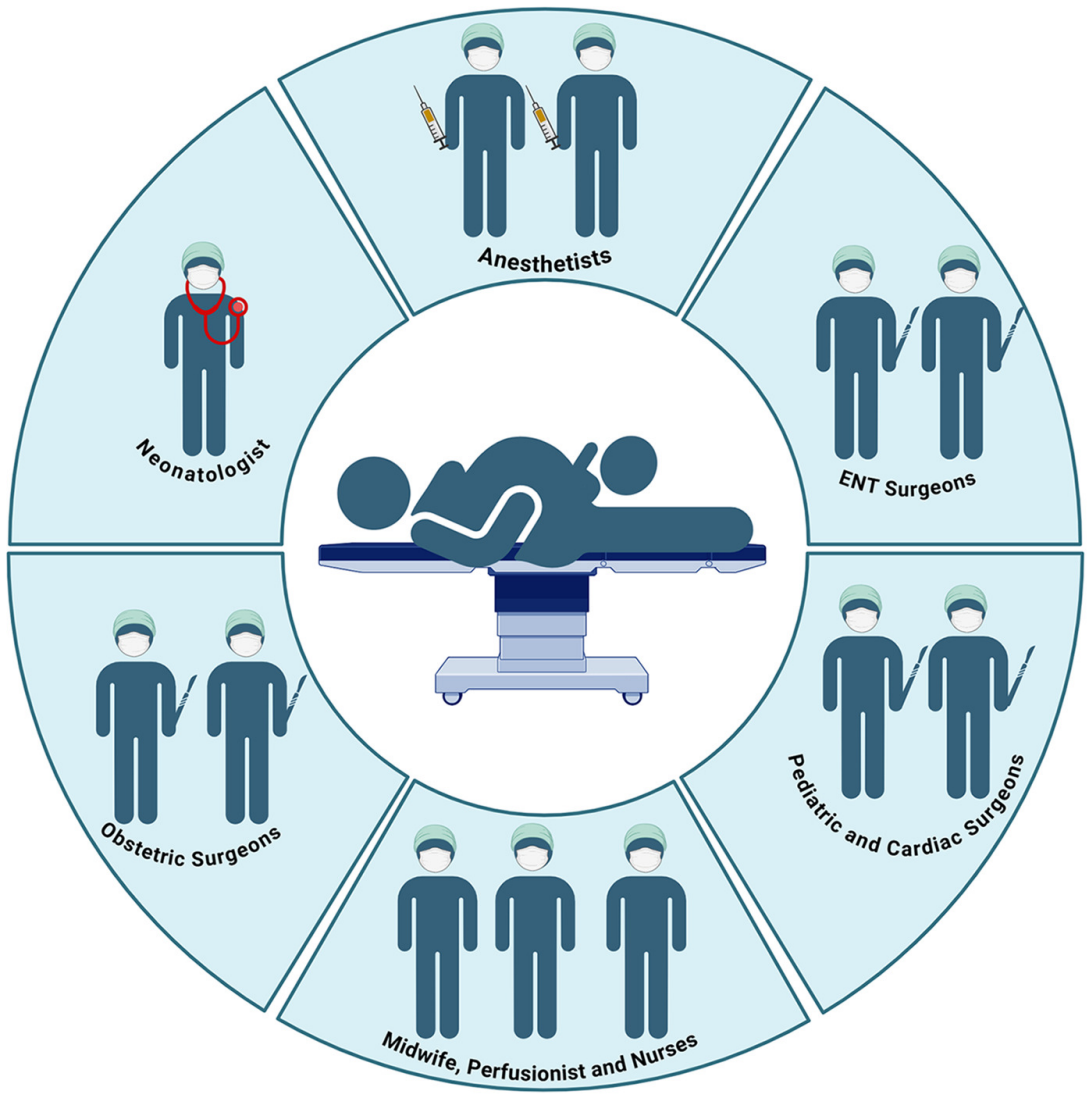
The complexity of the operating room during the EXIT procedure and the professional roles involved. Created in https://BioRender.com.

**Figure 5:**
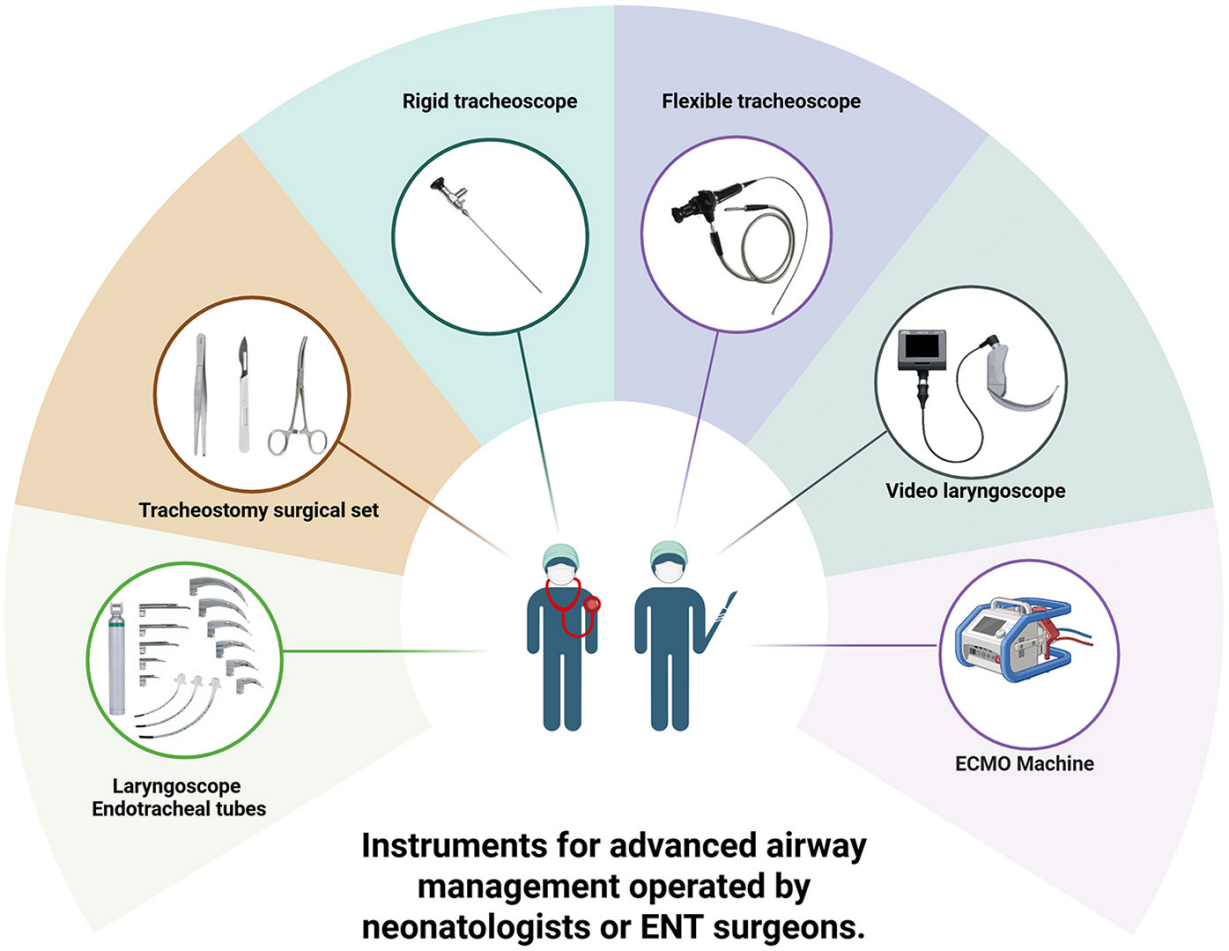
Instruments for advanced airway management operated by neonatologists or ENT surgeons. Created in https://BioRender.com.

The efficacy of the EXIT procedure is significantly influenced by meticulous multidisciplinary coordination and comprehensive preparation of the operating theater (OT) (140). These guarantee comprehensive patient care from preoperative planning through postoperative management (112). Essential team members include obstetricians, pediatric surgeons, anesthesiologists, neonatologists, otolaryngologists, specialized nursing staff, and, when necessary, cardiac surgeons and perfusionists to support ECMO (Figure 4) (10, 37, 70). Conducting a detailed preoperative briefing that includes a simulation of procedural steps enhances clarity of roles and preparedness for potential emergencies (120). The OT must be equipped with essential tools for neonatal resuscitation and intubation, capnography, sterile surgical instruments, uterine relaxants, and blood products to manage maternal hemorrhage effectively (6). Establishing parallel surgical fields for both neonatal and maternal care is necessary, and a secondary operating room or adjacent resuscitation area must be prepared to address any complications that may arise in either patient (141). Adequate team preparation through simulations and training is essential for ensuring safe and efficient procedures, consequently leading to improved outcomes for both the infant and the mother (78, 142–145). Neonatologists and otolaryngologists should receive training in advanced airway management techniques (Figure 5). Regular rehearsals and tailored strategies advance team synchronization, enhance communication, and mitigate risks, thus optimizing patient care (146, 147). This integrative approach emphasizes the critical importance of collaboration and simulation within the EXIT context, particularly for neonates necessitating complex, high-stakes interventions (123). Furthermore, three-dimensional modeling has proven instrumental in airway planning, family counseling, and perinatal management, especially in intricate clinical scenarios (148).

## 2 Conclusions

The EXIT procedure can be considered safe when conducted in specialized centers by a skilled multidisciplinary team. Despite inherent risks, it is crucial to address complex fetal anomalies. Maternal and fetal risks include bleeding, procedural complications, and neonatal mortality, emphasizing the need for careful case selection and expert execution. Ongoing research is essential to improve techniques, enhance safety, and broaden the procedure's applications for better outcomes.
